# Down-regulation of microRNA-144 in air pollution-related lung cancer

**DOI:** 10.1038/srep14331

**Published:** 2015-09-23

**Authors:** Hong-Li Pan, Zhe-Sheng Wen, Yun-Chao Huang, Xin Cheng, Gui-Zhen Wang, Yong-Chun Zhou, Zai-Yong Wang, Yong-Qing Guo, Yi Cao, Guang-Biao Zhou

**Affiliations:** 1 State Key Laboratory of Membrane Biology, Institute of Zoology, Chinese Academy of Sciences & Graduate School of the University of Chinese Academy of Sciences, Beijing 100101; 2 Department of Thoracic Surgery, the Cancer Hospital, Sun Yat-Sen University, Guangzhou 510060; 3 Department of Thoracic Surgery, the Third Affiliated Hospital of Kunming Medical University (Yunnan Tumor Hospital), Kunming 650106, China; 4 Department of Thoracic Surgery, China-Japan Friendship Hospital, Beijing 100029; 5 Laboratory of Molecular and Experimental Pathology, Kunming Institute of Zoology, Chinese Academy of Sciences, Kunming 650223, China

## Abstract

Air pollution has been classified as a group 1 carcinogen in humans, but the underlying tumourigenic mechanisms remain unclear. In Xuanwei city of Yunnan Province, the lung cancer incidence is among the highest in China, owing to severe air pollution generated by the combustion of smoky coal, providing a unique opportunity to dissect lung carcinogenesis. To identify abnormal miRNAs critical for air pollution-related tumourigenesis, we performed microRNA microarray analysis in 6 Xuanwei non-small cell lung cancers (NSCLCs) and 4 NSCLCs from control regions where smoky coal was not used. We found 13 down-regulated and 2 up-regulated miRNAs in Xuanwei NSCLCs. Among them, miR-144 was one of the most significantly down-regulated miRNAs. The expanded experiments showed that miR-144 was down-regulated in 45/51 (88.2%) Xuanwei NSCLCs and 34/54 (63%) control region NSCLCs (p = 0.016). MiR-144 interacted with the oncogene *Zeb1* at 2 sites in its 3′ untranslated region, and a decrease in miR-144 resulted in increased Zeb1 expression and an epithelial mesenchymal transition phenotype. Ectopic expression of miR-144 suppressed NSCLCs *in vitro* and *in vivo* by targeting Zeb1. These results indicate that down-regulation of miR-144 is critical for air pollution-related lung cancer, and the miR-144-Zeb1 signalling pathway could represent a potential therapeutic target.

Lung cancer is the most common cause of death from cancer worldwide and is estimated to have been responsible for nearly one in five deaths (1.59 million deaths, 19.4% of the total) in 2012^1^. Cigarette smoke is a major cause of lung cancer^2^, and indoor^3^ and outdoor^4^ air pollution have been classified as group 1 carcinogens in humans by the International Agency for Research on Cancer (IARC) of the World Health Organization. Anthropogenic particulate matter (PM) smaller than 2.5 μm in diameter (PM_2.5_) is associated with 220,000 lung cancer mortalities annually^5^, and is estimated to have cause 3.7 million premature deaths worldwide in 2012^6^. However, the carcinogenic mechanism of air pollution remains unclear.

In Xuanwei City of the Yunnan Province, the lung cancer mortality rate is among the highest in China, owing to severe air pollution produced by the combustion of smoky coal^7^,^8^,^9^. Residents in this city used smoky coal in unvented indoor firepits for domestic cooking and heating until the 1970s. These processes released high concentrations of PM_10_ and PM_2.5_, which contained high concentrations of polycyclic aromatic hydrocarbons (PAHs) including benzo(a)pyrene (BaP) and polar compounds that are highly mutagenic^9^. A reduction in lung cancer morbidity was noted in the 1990s after stove improvements were introduced in central Xuanwei, supporting the association between air pollution and lung cancer^10^. The IARC monograph cited the findings in Xuanwei and classified indoor emissions from household combustion of coal as “carcinogenic to humans (Group 1)”^3^. The population in these highly polluted regions (HPR) lends a unique opportunity to dissect carcinogenesis that is specifically related to air pollution. We utilized this opportunity by systematically analyzing the abnormalities in the cancer genomes, genome-wide DNA methylation, non-coding RNAs (miRNAs and lncRNAs), and inflammatory factors, in patients from this region. Alterations found in HPR lung cancers were tested in patients from control regions (CR) where smoky coal was not used to compare the difference in the carcinogenic mechanism between HPR and CR lung cancer. Here, we report our results in the assessment of the expression of miRNAs in HPR non-small cell lung cancers (NSCLCs).

## Results

### Identification of aberrant miRNAs in Xuanwei NSCLCs

**Figure 1:**
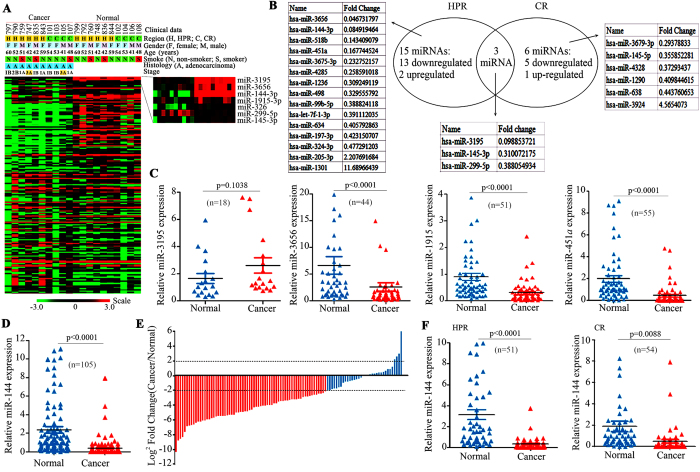
Identification of down-regulated miR-144 in HPR and CR NSCLCs. (**A**) MiRNA expression profiles of tumour and adjacent normal lung tissues from 10 NSCLCs. Left, data were organized according to the expression levels of individual miRNAs. Right, heat map showing 7 representative miRNAs down-regulated in the tumour samples. (**B**) Differentially expressed miRNAs in HPR compared to CR NSCLCs. (**C**) The expression of miR-3195, −3656, −1915-3p, and −451a in tumour samples and their paired normal lung tissues. The numbers of patients tested are shown in parentheses. (**D**, **E**) The expression of miR-144 in tumour samples and their counterpart normal lung tissues (**D**). MiR-144 expression was frequently decreased in cancer tissues (**E**). A log2 fold change greater than +2 or less than −2 was considered to be a significant up-regulation or down-regulation, respectively. (**F**) The expression of miR-144 in tumour samples and paired normal lung tissues from HPR and CR NSCLCs.

Tumour tissues and adjacent normal lung tissues were obtained with informed consent from 105 patients (51 from HPR and 54 from CR) with previously untreated lung adenocarcinoma (AD) or squamous cell carcinoma (SCC) (Table 1). Firstly, we performed miRNA microarray analysis for ten NSCLCs (6 from HPR and 4 from CR; Fig. 1A) and compared the expression of the miRNAs in tumour samples to that in their paired normal lung tissues to identify abnormal miRNA expression patterns. In total, we found 23 down-regulated and 7 up-regulated miRNAs in the 10 patients (Fig. 1A and Table 2). MiR-3195, miR-3656 and miR-144-3p (hereafter, miR-144) were the 3 most significantly down-regulated miRNAs (Fig. 1 and Table 2). We compared miRNA expression profiles between HPR and CR patients and found 21 miRNAs that were differentially expressed in HPR patients compared to CR cases. These included 13 down-regulated and 2 up-regulated miRNAs that were seen only in HPR and 5 down-regulated and 1 up-regulated miRNAs that were detected only in CR patients. Three miRNAs were down-regulated in NSCLCs from both regions (Fig. 1B).

To verify the results of the miRNA microarrays, we tested the expression of miR-3195, miR-3656, miR-144, miR-1915, and miR-451a in additional NSCLCs from HPR and CR. We found that the expression of miR-3195 in 18 NSCLC tumor samples did not differ from their paired normal lung tissues (Fig. 1C). The expression of miR-3656, miR-1915, and miR-451a was tested in 44 to 55 NSCLCs, and the results showed that these miRNAs were down-regulated in tumour samples compared to paired normal lung tissues (Fig. 1C). Notably, the expression of miR-144 was decreased by at least 4-fold in 68/105 (64.8%) NSCLCs compared to matched adjacent normal lung samples (Fig. 1D, E).

### Down-regulation of miR-144 is frequently seen in NSCLCs from HPR

We found that the expression of miR-144 was much lower in tumour samples than paired adjacent normal lung tissues in 45/51 (88.2%) and 34/54 (63%) NSCLCs from HPR and CR (Fig. 1F), respectively. The down-regulation of miR-144 in tumor samples was more frequently seen in HPR than CR NSCLCs (p = 0.016), indicating the association between air pollution and low miR-144 expression (Table 1).

To test whether air pollutants could down-regulate miR-144, several PAH compounds including benzo(a)pyrene (BaP), benzo(a)pyrene diol epoxide (BPDE), dibenzo[a, h]anthracene (DBA), and benzo[g, h, i]perylene (BzP) as well as the tobacco-specific carcinogen nicotine-derived nitrosamine ketone (NNK) were used to treat normal human bronchial epithelial 16HBE cells^11^ for 2 (Fig. S1A) or 15 (Fig. S1B) days. We showed that treatment of the cells with these compounds at concentrations of 0.25 and 0.5 μM for up to 15 days did not perturb the expression of miR-144. Therefore, isolation of other carcinogens and the assessment of the effects of these compounds on the expression of miR-144 warrant further investigation.

### Effects of miR-144 on NSCLC cell migrations

**Figure 2:**
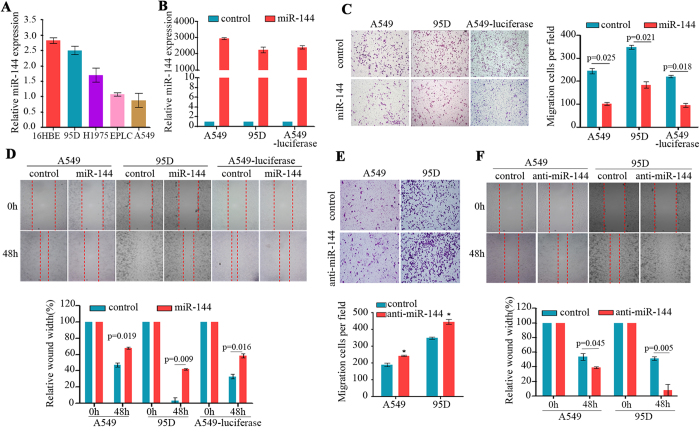
MiR-144 suppresses cell migration *in vitro*. (**A**) miR-144 expression is reduced in lung cancer cell lines compared to the immortalized normal human bronchial epithelial cell line 16HBE. (**B**) MiR-144 was increased in A549 and 95D cells after transfection of a miR-144 mimic. MiR-144 expression was determined by qRT-PCR 48 h after transfection and was calculated as the fold change relative to the miR-control. U6 served as an internal control. (**C**) MiR-144 overexpression decreased the number of migrated cells in the transwell assay. Cells were seeded into transwell chambers. After 24 h, migrated cells stained with 0.1% crystal violet were counted. Data represent the means ± SD from three independent experiments. (**D**) Inhibition of cell migration by the miR-144 mimic was confirmed by the wounding healing assay. Data represent the means ± SD from three independent experiments. (**E**) Inhibition of miR-144 by anti-miR-144 enhanced cell migration in the Transwell assay. *, p<0.05. (**F**) Anti-miR-144 treatment enhanced cell migration in the wounding healing assay.

We analysed the expression of miR-144 in human lung cancer cell lines by quantitative RT-PCR and found that the expression of this miRNA was low in the lung cancer cell lines 95D, H1975, EPLC, and A549, whereas 16HBE cells had a relatively high expression level of miR-144 (Fig. 2A). To determine whether miR-144 was a putative tumour suppressor, an miR-144 mimic was transfected into A549 and 95D cells by using Lipofectamine or into A549-luciferase cells by using lentivirus-mediated cell transfection (Fig. 2B), and cell migration assays were performed. By transwell and wound healing assays, we found that miR-144 dramatically suppressed the migration abilities of the cells (Fig. 2C, D). In contrast, suppression of miR-144 by using an miRNA inhibitor enhanced cell migration in both of the lung cancer cell lines (Fig. 2E, F).

### MiR-144 directly targets *Zeb1* by interacting with its 3′-untranslated regions (UTRs)

**Figure 3:**
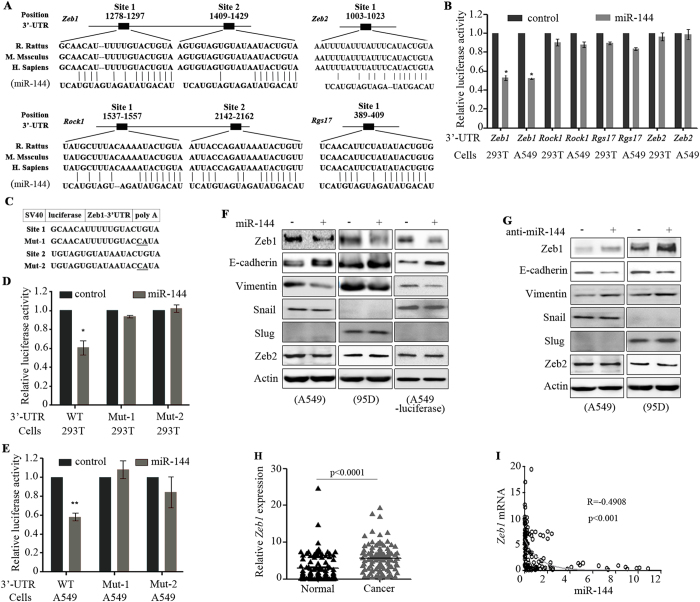
MiR-144 directly targets Zeb1. (**A**) *Zeb1*, *Zeb2*, *Rock1*, and *Rgs17* are targets of miR-144. Predicted duplex formation between *Zeb1*-3′-UTR, *Zeb2*-3′-UTR, *Rock1*-3′UTR, *Rgs17*-3′UTR and miR-144. (**B**) Luciferase assays in 293T and A549 cells transfected with a luciferase reporter controlled by the indicated elements. (**C**) Sequences of wild-type and mutant target sites for miR-144 in the *Zeb1*-3′ UTR. Two point mutations (underlined) predicted to abolish miRNA-mRNA binding were introduced into the miR-144 recognition region. (**D**, **E**) Luciferase reporter assays were performed in 293T (**D**) and A549 (**E**) cells co-transfected with the miR-control or miR-144 mimic (miR-144) together with the luciferase gene driven by the wild-type or mutant *Zeb1*-3′UTR sequences. The normalized luciferase activity in the control group was set as the relative luciferase activity. Each bar represents the mean ± S.D. for triplicate experiments. *P* values were determined by Student’s t-test. *p < 0.05; **p < 0.01. (**F**, **G**) The cells were transfected with the miR-control or miR-144 mimic (**F**) or anti-miR-144 (**G**) at a final concentration of 100 nM. The expression of the indicated proteins was determined by immunoblotting. (**H**) The expression of *Zeb1* in tumour samples and their paired normal lung tissues from the 105 NSCLCs. (**I**) The correlation between *Zeb1* and miR-144 expression was evaluated by Pearson correlation analysis. R and p-values are shown.

Next, we investigated the targets of miR-144. Based the bioinformatics analysis using TargetScan (http://www.targetscan.org/), Miranda (http://www.microrna.org/microrna/home.do) and microcosm (http://www.ebi.ac.uk/enright-srv/microcosm/htdocs/targets/v5/), four genes (*Zeb1*, *Zeb2*, *Rgs17*, and *Rock1*) were predicted to be targeted by miR-144. *Zeb1* and *Rock1* had 2 potential interacting sites, while *Zeb2* and *Rgs1* had 1 potential interacting site with miR-144 (Fig. 3A). Then, we performed firefly luciferase reporter assays using the 3′-UTRs of *Zeb1* (*Zeb1*-3UTR), *Zeb2* (*Zeb2*-3UTR), *Rgs17* (*Rgs17*-3UTR), and *Rock1* (*Rock1*-3UTR) and found that the relative luciferase activity of the reporter that contained the *Zeb1*-3UTR was decreased by 47.0% in HEK293T cells and 47.5% in A549 cells after co-transfection of the miR-144 mimic (Fig. 3B). Conversely, co-transfection of the miR-144 mimic did not perturb *Zeb2*, *Rgs17* or *Rock1* reporter activity (Fig. 3B). Furthermore, mutations in potential interaction sites 1 and 2 of *Zeb1* (Fig. 3C) abrogated miR-144-induced suppression of *Zeb1*-luciferase in the 293T (Fig. 3D) and A549 cells (Fig. 3E).

To further determine whether miR-144 affected endogenous *Zeb1* expression, A549 and 95D cells were transfected with a miR-control or miR-144 mimic. In cells transfected with the miR-144 mimic, Zeb1 expression was down-regulated (Fig. 3F) at the protein level, whereas its target E-cadherin^12^ was up-regulated and Vimentin was down-regulated (Fig. 3F). In contrast, transfection of anti-miR-144 into the cells resulted in up-regulation of the Zeb1 protein (Fig. 3G), suggesting that Zeb1 could be a target gene of miR-144. However, the expression of Zeb2 was not affected by miR-144 overexpression or silencing (Fig. 3B, F, G). Moreover, the transcription factors Snail and Slug which play important roles in the epithelial mesenchymal transition (EMT) through transcriptional activation of Zeb1^13^,^14^, were not affected by miR-144 overexpression or silencing (Fig. 3F, G), confirming the results that Zeb1 has no effect on the expression of Snail and Slug^15^.

### The expression of *Zeb1* in HPR NSCLCs

We tested the expression of *Zeb1* in the 105 NSCLCs, and found that *Zeb1* expression in tumour samples was much higher than in paired normal lung tissues (Fig. 3H). The expression of *Zeb2* was also elevated in the patients’ tumour samples (Fig. S2), which was consistent with previous reports^16^. Using Pearson correlation analysis, we evaluated the potential correlation between miR-144 and *Zeb1* expression and found that miR-144 was inversely associated with *Zeb1* expression (Fig. 3I).

### Zeb1 inhibition recapitulates the tumour suppressing effect of miR-144

**Figure 4:**
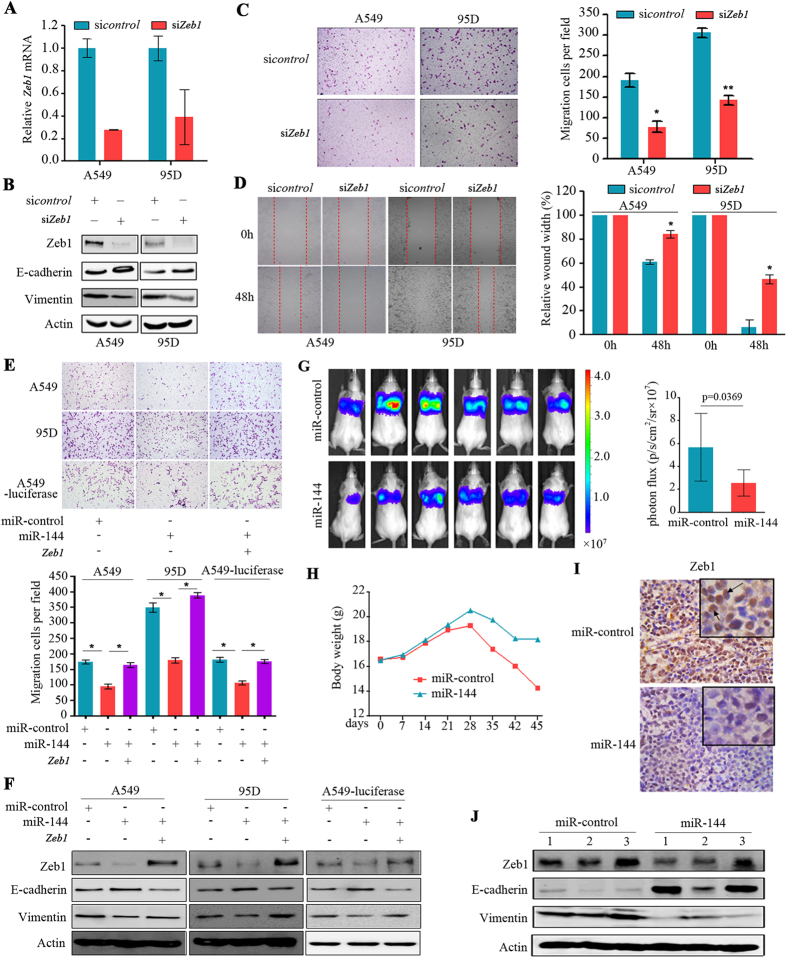
Inhibition of Zeb1 mediates the tumour suppressor functions of miR-144. (**A**, **B**) Endogenous Zeb1 expression was efficiently repressed by siRNA. A549 and 95D cells were transfected with non-specific siRNA control (sicontrol) or Zeb1-specific siRNA (siZeb1) for 48 h. The expression of Zeb1 were measured by qRT-PCR (**A**) and Western blotting (**B**). Inhibition of Zeb1 expression led to up-regulation of E-cadherin and down-regulation of Vimentin (**B**). (**C**) Inhibition of Zeb1 expression reduced the number of migrated cells in the Transwell assay. (**D**) Inhibition of cell migration by siZeb1 was confirmed by the wounding healing assay. Data represent the means ± SD from three independent experiments. (**E**) Ectopic expression of *Zeb1* rescued the inhibition of cell migration associated with the re-expression of miR-144 in A549, 95D, and A549-luciferase-miR-144 cells. Transwell assays were performed after the co-transfection of the miR-144 mimic and the plasmid containing *Zeb1*. (**F**) Zeb1 re-expression abrogated miR-144-induced up-regulation of E-cadherin and down-regulation of Vimentin. (**G**–**J**) Six-week-old SCID Beige female mice were administered with 1 × 10^6^ A549-luciferase cells stably expressing miR-144 or the miR-control via the tail veins. (**G**) One month later, tumours in the lungs were surveyed with the IVIS Spectrum Imaging System. (**H**) Body weights of the mice were measured. (**I**) Immunohistochemistry (IHC) staining of Zeb1 in lung tissues. (**J**) The expression of Zeb1, E-cadherin, and Vimentin was detected by Western blotting.

To evaluate whether down-regulation of *Zeb1* was involved in the miR-144-induced suppression of cell migration, A549 and 95D cells were transfected with a *Zeb1*-specific siRNA (siZeb1) that caused down-regulation of Zeb1 at the mRNA (Fig. 4A) and protein (Fig. 4B) levels. Silencing of Zeb1 led to up-regulation of E-Cadherin and down-regulation of Vimentin (Fig. 4B). Furthermore, we found that silencing of Zeb1 significantly suppressed the cell migration of A549 and 95D cells in the transwell (Fig. 4C) and wound-healing (Fig. 4D) assays. Whereas transfection of miR-144 suppressed the migration of cancer cells, co-transfection of *Zeb1* attenuated this effect (Fig. 4E). Consistent with this, transfection of miR-144 into the cells caused down-regulation of Zeb1 and Vimentin and up-regulation of E-Cadherin, whereas ectopic expression of Zeb1 partially reversed these effects (Fig. 4F).

### MiR-144 inhibits cancer progression *in vivo*

To test the *in vivo* tumour suppressing effect of miR-144, either the miR-control or miR-144 was stably transfected into A549-luciferase cells, which were then injected into the tail veins of SCID Beige mice. We showed that, compared to mice injected with miR-control-transfected cells, mice injected with miR-144-A549-luciferase cells showed a three-fold reduction in lung tumour volume detected by the IVIS Spectrum Imaging System (Fig. 4G). The body weights of the mice harboring miR-control-A549-luciferase cells decreased rapidly, whereas the body weights of mice bearing miR-144-A549-luciferase cells decreased gradually (Fig. 4H), reflecting the tumour burden and progression. The immunohistochemistry (Fig. 4I) and Western blot (Fig. 4J) assays showed that both Zeb1 and Vimentin were down-regulated and E-Cadherin was up-regulated in tumours harvested from mice inoculated with the miR-144-A549-luciferase cells.

## Discussion

MiRNAs are small non-coding RNAs that post-transcriptionally mediate the expression of their target genes by perfect or imperfect binding to the 3′-UTR^17^, the 5′-UTR^18^, or the open reading frame of the target mRNAs^19^, thereby causing mRNA degradation or inhibition or enhancement of mRNA translation. It has been estimated that miRNAs regulate more than 60% of human genes^20^. MiRNAs participate in diverse biological processes, including cell proliferation, apoptosis, migration and invasion^21^. Some miRNAs have been shown to be involved in the tumourigenesis of lung cancer, and miRNA expression profiles are diagnostic and prognostic markers of lung cancer^22^. For instance, microRNA-135b promotes lung cancer metastasis by targeting tumour suppressor LZTS1 and multiple key components in the Hippo pathway such as LATS2, β-TrCP and NDR2^23^. MicroRNA-193a-3p and −5p function as tumour suppressors and inhibit the metastasis of lung cancer by down-regulating the ERBB4/PIK3R3/mTOR/S6K2 signalling pathway^24^. MiR-21^25^, miR-17-92^26^ and miR-196a^27^ are up-regulated and function as oncogenes, whereas let-7^28^, miR-194^29^ and miR-126^30^ suppress carcinogenesis. However, abnormalities in miRNAs in air pollution-related lung cancer have not been investigated.

PAHs, the main carcinogens in HPR^9^, are ubiquitous environmental pollutants that are generated primarily through incomplete combustion of carbon-containing materials. In cellular or animal models, PAHs down-regulate miR-34c^31^, miR-21, miR-221, miR-222, and miR-429^32^, and up-regulate miR-34a^33^,^34^, miR-181a, miR-181b, and miR-181d^35^. Deng *et al.*^36^ performed a study in healthy male coke oven workers to identify miRNAs associated with PAH exposure, and found that urinary 4-hydroxyphenanthrene and/or plasma BPDE–Alb adducts were associated with lower miR-24-3p, miR-27a-3p, miR-142-5p, and miR-28-5p expression. Urinary 1-hydroxynaphthalene, 2-hydroxynaphthalene, 2-hydroxyphenanthrene, and the sum of monohydroxy-PAHs were associated with higher miR-150-5p expression. Here, we performed miRNA microarrays to screen for abnormal miRNAs in air pollution-related lung cancer, using samples from a unique HPR and found that miR-144 and its family member miR-451^37^ were significantly down-regulated in the patients. We expanded this observation and reported that miR-144 was down-regulated in 45/51 (88.2%) HPR NSCLCs, whereas in CR patients miR-144 was suppressed in 34/54 (63%) tumour samples (p = 0.016). Our results provided the first evidence of association between a miRNA and air pollution-related lung cancer. However, treatment of 16HBE cells with the carcinogens BaP, BPDE, BzP, DBA and NNK for up to 15 days did not result in the down-regulation of miR-144, suggesting that other carcinogens may be responsible for the down-regulation of this miRNA; therefore, investigations are warranted to uncover the carcinogen(s).

MiR-144 can modulate TRAIL-induced apoptosis by targeting caspase-3^38^. Down-regulation of miR-144 is associated with colorectal cancer progression via activation of the mTOR signalling pathway^39^. MiR-144 inhibits NSCLC cell growth and induces apoptosis by down-regulating ZFX^40^, and re-establishing miR-144 in gastric cancer restores the chemosensitivity^41^. We showed that *Zeb1*, a transcriptional repressor^42^ that inhibits E-cadherin and promotes EMT and metastasis^12^,^43^, is a target of miR-144. MiR-144 interacts with *Zeb1* via two sites in *Zeb1*’s 3′-UTR, and mutations in these sites abrogate miR-144’s repression of Zeb1 functions (Fig. 3), a result in agreement with previous reports^44^,^45^. In lung cancer, the expression of Zeb1 is up-regulated^46^, whereas knockdown of Zeb1 results in dramatic growth inhibition^47^. MiR-144 could also bind *Zeb2* at positions 1003-1023 in its 3′-UTR (Fig. 3A), but in NSCLC cells overexpression or knockdown of miR-144 had no effect on the expression of Zeb2 at the mRNA and protein levels (Fig. 3B, F, G). In NSCLCs, miR-200b and miR-200c, which are able to bind *Zeb2* at positions 1015-1036 of its 3′-UTR, were up-regulated^48^,^49^, and may therefore antagonize the effects of miR-144 on *Zeb2*. Hence, our results indicated that miR-144 is an important tumour suppressor that is inactivated during malignant transformation, and the miR-144-Zeb1 signal pathway could represent a rational therapeutic target.

## Methods

### Patients and tissue samples

Use of the samples was approved by the Institutional Review Board of the Institute of Zoology, Chinese Academy of Sciences, and the local research ethics committees of all participating hospitals. The methods were performed in accordance with the approved guidelines. Tumour tissues and adjacent normal lung tissues were obtained with informed consent from 105 patients with previously untreated lung adenocarcinoma (AD) or squamous cell carcinoma (SCC) at local hospitals. The diagnosis of lung cancer was confirmed by at least 3 pathologists, and the HPR patients enrolled met the following criteria: (1) patients were residents of Xuanwei where the smoky coal was used; (2) patients resided in their communities and never stayed in other regions for a long period of time (6 months or more); (3) patients had previously untreated primary lung cancer; and (4) patients’ tissue samples were taken at the time of surgery and quickly frozen in liquid nitrogen. The tumour samples contained a tumour cellularity of greater than 60% and the matched control samples had no tumour content. The clinical and pathological data for these patients are shown in Table 1 and Fig. 1A.

### Cell culture

The NSCLC cell lines A549 and 95D, human bronchial epithelial cell line 16HBE, and a human embryonic kidney cell line HEK293T were cultured in DMEM (Hyclone, Logan, UT, USA) medium supplemented with 10% foetal bovine serum (Hyclone). The cells were treated with PAHs (BaP, BPDE, DBA, and BzP) or the tobacco specific carcinogen NNK. Cell viability was estimated by trypan blue dye exclusion analysis, and cell proliferation was measured by the MTT assay^50^.

### MiRNA microarray and quantitative RT-PCR

Total RNA was isolated using TRIzol (Invitrogen, Frederick, MD, USA) and the miRNeasy mini kit (Qiagen, Hilden, Germany), quantitated by using the NanoDrop 1000 (Thermo Scientific, Wilmington, DE, USA), labelled by using the miRCURY™ Hy3™/Hy5™ Power labelling kit (Exiqon, Vedbaek, Denmark) and hybridized onto the miRCURY™ LNA Array (v.16.0; Exiqon). Following the washing steps, the slides were scanned using the Axon GenePix 4000B microarray scanner (Axon Instruments, Foster City, CA, USA), and images were imported into the GenePix Pro 6.0 software (Axon Instruments) for grid alignment and data extraction. All expressed data were normalized using the Median normalization method^51^, and significantly differentially expressed miRNAs were identified through Volcano Plot filtering. Quantitative RT-PCR analysis for miR-3195, −3656, −144, −451a and −1915-3p was performed with a miScript SYBR Green PCR Kit (Qiagen). The expression of related genes was measured by qPCR with SYBR^®^ Green Real time PCR Master Mix (Takara Biotechnology, Dalian, China). The fold changes in mRNA expression were calculated using the 2^−ΔΔCt^ method^52^. The primers used for quantitative RT-PCR are as follows: *Zeb1*, forward, 5′-ATGCACAACCAAGTGCAGAAGA-3′ and reverse, 5′-TTGCCTGGTTCAGGAGAAGATG-3′; *Zeb2*, forward, 5′- CAAGAGGCGCAAACAAGC-3′ and reverse, 5′- GGTTGGCAATACCGTCAT-3′; *E-cadherin*, forward, 5′-TGCCCAGAAAATGAAAAAGG-3′ and reverse, 5′-GTGTATGTGGCAATGCGTTC-3′; *Vimentin*, forward, 5′-GAGAACTTTGCCGTTGAAGC-3′ and reverse, 5′-GCTTCCTGTAGGTGGCAATC-3′; and *GAPDH*, forward, 5′-GAGTCAACGGATTTGGTCGT-3′ and reverse, 5′-GACAAGCTTCCCGTTCTCAG-3′.

### Wound-healing assay and *in vitro* migration assay

For the wound-healing assay, the cells (4 × 10^5^/wells) were seeded into six-well plates and transfected the next day with a miR-144 mimic or a miR-control at a final concentration of 100 nM. Twenty-four hours after transfection, wounds were created in the cell monolayer using a p200 micropipette tip. The healing process was followed for the next 48 hours. For the transwell assay, transwell inserts (6.5 mm diameter and 8 μm pore size; Corning Inc., Corning, NY, USA) were rehydrated by adding serum-free medium for at least 1 hour. The cells transfected with the miR-144 mimic in serum-free medium were seeded (2 × 10^4^ cells) into the inserts (the upper chamber). Complete medium was used as a chemoattractant in the bottom chamber. After 24 hours of migration, the cells in the upper surface of the insert membrane were removed by wiping with a cotton swab, and cells in the lower surface were fixed with methanol, stained with crystal violet, and counted by microscopy.

### Luciferase report assays

The miR-144 binding site-containing 3′ UTR fragment of *Zeb1* was amplified and cloned into the modified pGL3-luciferase vector. Mutations in the *Zeb1* 3′-UTR were generated by using the QuikChange Site-Directed Mutagenesis Kit (Stratagene, La Jolla, CA, USA). The miR-144 mimic (5′-UACAGUAUAGAUGAUGUACU-3′), miR-control (5′-UUCUCCGAACGUGUCACGUTT-3′), anti-miR-144 (5′-AGUACAUCAUCUAUACUGUA), anti-miR-control (CAGUACUUUUGUGUAGUACAA), si*Zeb1* (5′-UGAUCAGCCUCAAUCUGCATT-3′) and si-control (UUCUCCGAACGUGUCACGUTT) were synthesized by GenePharma (Shanghai, China). The cells were transfected with 0.5 μg of pGL3-luciferase vector and 50 nM of the miR-144 mimic or miR-control together with a Renilla plasmid using Lipofectamine 2000 (Invitrogen) according to the manufacturer′s instructions. Luciferase activities were measured using the Dual luciferase reporter assay system (Promega, Madison, WI, USA).

### Lentivirus-mediated cell transfection and transduction

Pre-miR-144 DNA sequences were amplified from human genomic DNA, subcloned into the EcoR I and BamH I sites downstream of the CMV promoter in the pCDH vector (kindly provided by Dr. Wanzhu Jin at the Institute of Zoology, Chinese Academy of Sciences), and verified by DNA sequencing. The primers used for the genomic PCR amplification of miR-144 were as follows: 5′-GCGCGAATTCGAGATCTTAACAGACCCTAGCTC-3′ (forward primer) and 5′-GCGCGGATCCGTGCCCTGGCAGTCAGTAGG-3′ (reverse primer). Infectious virus particles were harvested 48 hours after co-transfection of pCDH-miR-144 or pCDH with the lentivirus packing vector (psPAX2 and pMD2G) into HEK293FT cells. A549 cells were infected with lentiviruses in medium containing polybrene (8 mg/ml). One week after infection, the cells were sorted by flow cytometry.

### Western Blotting

Cells were lysed in RIPA buffer supplemented with a protease inhibitors cocktail (Sigma, St. Louis, MO, USA). Proteins (20 μg) were subjected to 10–15% SDS-PAGE, electrophoresed and transferred on to a nitrocellulose membrane. After blocking with 5% non-fat milk in Tris-buffered saline, the membrane was washed and incubated with the indicated primary and secondary antibodies and detected using the Luminescent Image Analyser LSA 4000 (GE, Fairfield, CO, USA).

### Animal studies

The animal studies were approved by the Institutional Review Board of the Institute of Zoology, Chinese Academy of Sciences. The methods were performed in accordance with the approved guidelines. Six-week-old SCID Beige mice were maintained under specific pathogen-free (SPF) conditions. A549-luciferase cells (1 × 10^6^) stably expressing miR-144 or the miR-control were injected into the lateral tail veins of the mice (6 per group). After 30 days, the tumours were monitored with the IVIS Spectrum Imaging System (Caliper Life Sciences; Hopkinton, MA, USA). For the immunohistochemistry (IHC) assay, sections were fixed in formalin and embedded in paraffin, incubated with primary antibodies overnight, and then incubated with anti-rabbit IgG secondary antibodies. Detection was conducted using 3, 3′-diaminobenzidine (DAB, Zhongshan Golden Bridge Biotechnology Co., Ltd, Beijing, China) and haematoxylin.

### Statistical analysis

Experimental data were presented as the mean ± SD of three independent experiments. The differences between groups were estimated using an independent two-tailed Student’s *t*-test (normal distribution data) or Wilcoxon rank sum test (non-normal distribution data), and the association between miR-144 expression and the Zeb1 level was analysed by Pearson correlation analysis. *P* values less than 0.05 were considered statistically significant in all cases.

## Additional Information

**How to cite this article**: Pan, H.-L. *et al.* Down–regulation of microRNA-144 in air pollution-related lung cancer. *Sci. Rep.* **5**, 14331; doi: 10.1038/srep14331 (2015).

## Supplementary Material

Supplementary Information

## Figures and Tables

**Table 1:** Baseline demographic characteristics of the patients.

**Characteristics**	**Case, n**	**miR-144 downregulation, n (%)**	***P* value**
Total	105	79 (75.2%)	
Regions			
HPR	51	45 (88.2%)	0.016
CR	54	34 (63.0%)	
Gender			
Male	62	45 (72.6%)	0.55
Female	43	34 (79.0%)	
Smoking: Total			
Smoker	44	31 (70.5%)	0.88
Non-smoker	61	48 (78.7%)	
*HPR*			
Smoker	20	16 (80.0%)	0.42
Non-smoker	31	29 (93.5%)	0.97
*CR*			
Smoker	24	15 (62.5%)	
Non-smoker	30	19 (63.3%)	
Age			
<60	58	43 (74.1%)	0.73
>=60	47	36 (76.6%)	
Histology			
Adenocarcinoma	74	54 (73.0%)	0.24
Squamous-cell carcinoma	29	23 (79.3%)	
Large cell neuroendocrine carcinoma	2	2 (100%)	
TNM Stage			
I	42	33 (78.6%)	0.90
II	24	17 (70.8%)	
III	28	19 (67.9%)	
IV	11	10 (90.9%)	

**Table 2:** The abnormal miRNAs found in the 10 NSCLCs.

**No.**	**Name**	**Mean of cancer group**	**Mean of normal group**	**Fold change (Cancer/Normal)**	***P*** **value**
1	hsa-miR-3195	0.270997195	2.73201341	0.099193215	8.32E-06
2	hsa-miR-3656	0.273645794	1.90825264	0.143401239	0.000934427
3	hsa-miR-144-3p	2.751036037	13.17806638	0.208758702	0.039456994
4	hsa-miR-1915-3p	0.296520317	1.195654589	0.247998309	0.048055479
5	hsa-miR-326	0.054826095	0.194955184	0.281224094	0.037585277
6	hsa-miR-299-5p	0.800828145	2.532885779	0.31617223	0.000172098
7	hsa-miR-145-3p	0.107968344	0.319009068	0.338449137	0.000609156
8	hsa-miR-523-3p	0.021139828	0.062039785	0.340746317	0.019238358
9	hsa-miR-3675-3p	0.047118694	0.132957511	0.354389108	0.000247249
10	hsa-miR-451a	6.818830466	18.25961551	0.37343779	0.042307196
11	hsa-miR-197-3p	0.029877596	0.078804482	0.379135742	0.000403023
12	hsa-miR-1236	0.082057424	0.21278583	0.385633872	0.011617197
13	hsa-miR-3685	0.112880187	0.279113338	0.404424195	0.020700161
14	hsa-miR-99b-5p	0.171215455	0.411854791	0.415718012	0.010689398
15	hsa-miR-30e-3p	0.382749671	0.907446162	0.421787745	0.039507452
16	hsa-miR-126-5p	1.370886525	3.186303353	0.430243568	0.046789093
17	hsa-miR-30a-5p	1.675253097	3.754874549	0.446154212	0.040593378
18	hsa-miR-498	0.533734468	1.190437108	0.448351672	0.001745869
19	hsa-miR-140-3p	0.49929489	1.098995113	0.454319481	0.034968225
20	hsa-let-7f-1-3p	0.05073319	0.111280358	0.455904266	0.005368303
21	hsa-miR-145-5p	1.753949676	3.837430966	0.457063513	0.025818814
22	hsa-miR-324-3p	0.0614656	0.124925257	0.492018997	0.033740127
23	hsa-miR-125a-5p	3.490882724	6.245785777	0.498918101	0.041502739
24	hsa-miR-519e-3p	0.353096683	0.175603687	2.010758941	0.038365716
25	hsa-miR-944	0.093106955	0.043903965	2.120695816	0.022455575
26	hsa-miR-20b-3p	0.183502017	0.063425287	2.893199626	0.019566651
27	hsa-miR-196a-3p	0.337349575	0.10896787	3.095862809	0.037159431
28	hsa-miR-489	0.098459716	0.02842752	3.463535211	0.029405297
29	hsa-miR-3924	0.24073172	0.054093821	4.450262823	0.011610159
30	hsa-miR-1301	0.066751035	0.006652014	10.03471077	0.029476776
