# Supplementary material for: Down-regulation of microRNA-144 in air pollution-related lung cancer
Source: Sci Rep. 2015 Sep 23;5:14331. doi: 10.1038/srep14331 (PMC4585805; doi:10.1038/srep14331)

**Supplementary information**

**Downregulation of microRNA-144 in air pollution-related lung cancer**

Hong-Li Pan^1*^, Zhe-Sheng Wen^2*^, Yun-Chao Huang^3*^, Xin Cheng^1^, Gui-Zhen Wang^1^, Yong-Chun Zhou^3^, Zai-Yong Wang^4^, Yong-Qing Guo^4^, Yi Cao^5^, Guang-Biao Zhou^1^

^1^State Key Laboratory of Membrane Biology, Institute of Zoology, Chinese Academy of Sciences & University of Chinese Academy of Sciences, Beijing 100101;

^2^Department of Thoracic Surgery, the Cancer Hospital, Sun Yat-Sen University, Guangzhou 510060;

^3^Department of Thoracic Surgery, the Third Affiliated Hospital of Kunming Medical University (Yunnan Tumor Hospital), Kunming 650106;

^4^Department of Thoracic Surgery, China-Japan Friendship Hospital, Beijing 100029;

^5^Laboratory of Molecular and Experimental Pathology, Kunming Institute of Zoology, Chinese Academy of Sciences, Kunming 650223, China.

**Supplementary figures and figure legends**

Figure S1. Effects of carcinogens on the expression of miR-144. (A) The expression of miR-144 in 16HBE cells treated with indicated carcinogens for 2 days. Detected by real-time RT-PCR. (B) The expression of miR-144 in 16HBE cells treated with indicated carcinogens for 15 days. Detected by real-time RT-PCR.


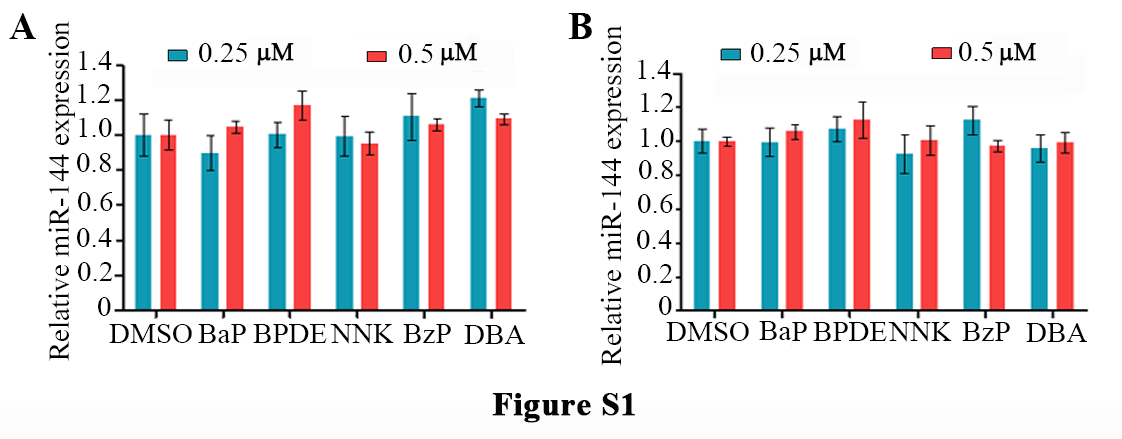


Figure S2. The expression of *Zeb2* in NSCLCs detected by real-time RT-PCR.


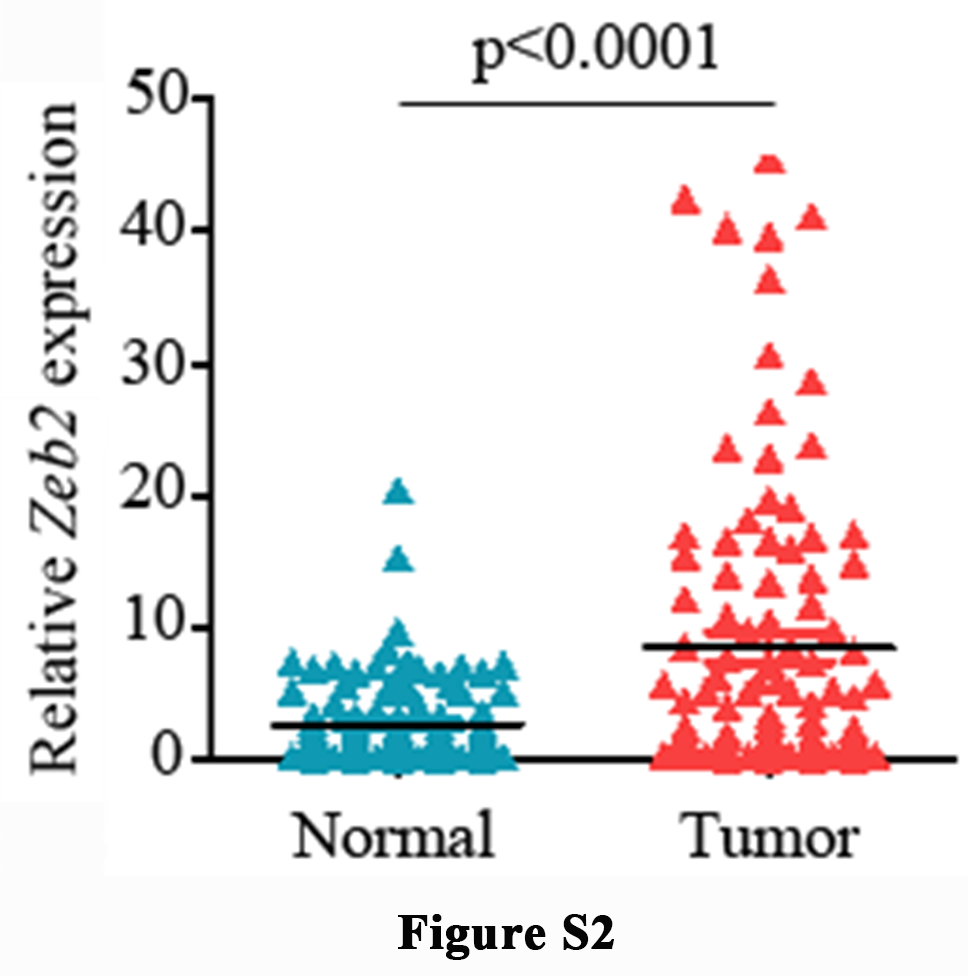

Supplement: Supplementary Information [file srep14331-s1.doc]
